# The Relationship Between the Presence of White Nails and Readmission Among Rural Older Admitted Patients: A Prospective Cohort Study

**DOI:** 10.7759/cureus.24297

**Published:** 2022-04-19

**Authors:** Ryuichi Ohta, Yoshinori Ryu, Chiaki Sano

**Affiliations:** 1 Community Care, Unnan City Hospital, Unnan, JPN; 2 Community Medicine Management, Shimane University Faculty of Medicine, Izumo, JPN

**Keywords:** hospital readmission, white nail, rural hospital, general medicine, family medicine, elderly patients

## Abstract

Background

Nail changes can indicate systemic changes within the body. According to previous studies, white nails, characterized by the whitening of the proximal nails with the absence of a lunula, were related to in-hospital mortality in rural community hospitals. Hence, this study aimed to investigate the relationship between the presence of white nails and readmissions among rural older patients who had previously been discharged from rural community hospitals.

Methodology

The relationship between white nails and readmissions among admitted older patients discharged from community hospitals was investigated. This was a single-center prospective study. All patients >65 years admitted from April 2020 to March 2021 and readmitted between April 2020 and June 2021 were included in the study. Upon admission, all patients’ nails were evaluated by trained evaluators for whitening. The presence of white nails was correlated with readmission using a multivariate logistic regression model.

Results

Approximately 28.7% of all participants were readmitted to the hospital during the study period; 41.1% had white nails, and 24.6% did not. Cox hazard model was used to analyze the relationship between readmission and white nails. Of all 637 participants, 24.8% had white nails. Significant variables for readmission were cancer (hazard ratio, HR = 1.52, p = 0.033), dementia (HR = 1.52, p = 0.037), heart failure (HR = 1.53, p = 0.033), home discharge (HR = 0.32, p ≤ 0.001), duration of previous hospitalization (HR = 0.99, p = 0.0026), and white nails (HR = 2.07, p ≤ 0.001).

Conclusions

White nails may be associated with readmission among previously admitted older patients. Identifying white nails in older admitted patients may enhance awareness of readmission risk; however, this needs further research.

## Introduction

Nail color changes can indicate various medical conditions. Therefore, assessing the medical conditions of patients based on nail conditions is essential. Nutritional conditions and chronic diseases affect nail conditions [1]. Nail color tends to change gradually, depending on the state of nail beds [2]. Therefore, changes in nail color may indicate chronic changes in the body [3]. For example, patients with heart failure, liver cirrhosis, or advanced age with poor nutrition tend to experience nail whitening. This is because the distal parts of the nail turn brown due to telangiectasis. This condition of the nail is called Terry’s nails [4]. Besides, conditions such as malnutrition and renal disease can cause nail whitening with brown distal parts due to melanin deposition, which is called Lindsay’s nails [4]. The mechanisms underlying Terry’s and Lindsay’s nails are not fully understood. Therefore, nail whitening assessment is accessible and valuable for effective patient care management.

A white nail can be defined as the disappearance of the lunula and whitening of the nail bed on any finger [5]. Nail whitening may appear due to chronic medical conditions. Detecting white nails can help predict the medical conditions of older patients. It can be used to assess the previous conditions of patients with acute symptoms because nail plates and bed conditions may not change as static physical findings [5]. A previous study showed that clinicians could predict the risk of death in older admitted patients with white nails [6]. In addition, the presence of white nails can indicate comorbidities and nutritional conditions that may influence morbidity and mortality [7].

Detecting white nails can be helpful for older admitted patients to assess their factors. Older patients tend to have multiple chronic diseases affecting their medical conditions. The conditions may modify specific physical examination findings [8]. In addition, aging can change the nail plate and bed conditions caused by the stiffness of arteries affecting the peripheral tissues [9]. Therefore, nail conditions can be examined easily such that healthcare professionals can check the presence of white nails in older patients during clinical visits.

The presence of white nails can indicate increased mortality risk and other trajectories, such as readmission, among older admitted patients. After discharge, instability of certain medical conditions can be related to hospital readmission [10]. To the best of the authors’ knowledge, no study has investigated the specific relationship between white nails and hospital readmission. By clarifying this relationship, the medical care team may better predict the risk of readmission and institute more intensive care for patients found to be at risk after discharge. As aging societies progress, the lack of healthcare resources may imply that checking nail conditions as part of a comprehensive assessment of an older patient is useful. This study aimed to investigate the relationship between white nails and readmissions among rural older patients who had previously been discharged from rural community hospitals.

## Materials and methods

Methods

This prospective cohort study included patients admitted to the Department of General Medicine in Unnan City Hospital. The patients were discharged from Unnan City Hospital during the study period.

Setting

Unnan City, located in the southeast of Shimane Prefecture, is one of the most rural cities in Japan. In 2020, the total population of Unnan was 37,638 (18,145 males and 19,492 females) at the time of the study, with 39% aged >65 years, a proportion expected to reach 50% by 2025 [10]. During this study, Unnan City Hospital had 281 beds comprising 160 acute care beds, 43 comprehensive care beds, 30 rehabilitation beds, and 48 chronic care beds. All patients were regularly followed up at Unnan City Hospital or other medical institutions in Unnan City from April 1, 2020, to June 30, 2021. All readmissions occurred at Unnan City Hospital [10].

Participants

All patients >65 years admitted to Unnan City Hospital from April 1, 2020, to March 31, 2021, were included in this study. In addition, any patient readmitted between April 1, 2020, and June 30, 2021, was included in the readmission group. Patients who lost fingers, had fungal nail infections in all fingers, had traumatic nail deformation, or died following admission were excluded.

Measurements

Primary Outcome

The primary outcome was readmission to the hospital with symptoms of acute disease. The time to readmission was measured as the number of days between the initial discharge and readmission. The reasons for readmission were extracted from the hospital’s electronic medical records.

Assessment of White Nails

**Figure 1:**
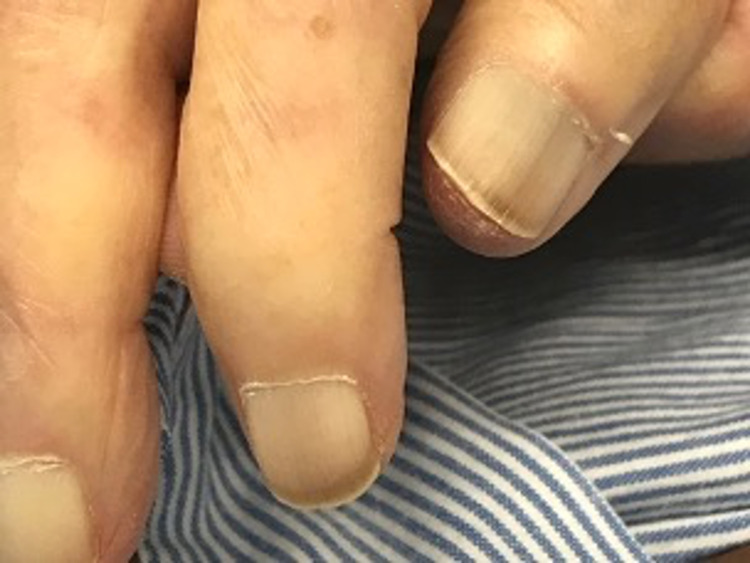
The training photograph of white nails.

The findings of white nails were characterized by a white appearance of the proximal nails with the absence of a lunula. Color changes in the distal region of nails were excluded from the assessment [5,6]. The nail lunula is eventually diminished in all fingers; however, the lunula of the first finger is often persistent, even in older individuals. Thus, the evaluation of the first fingers was excluded from the nail assessments. Upon admission, all patients’ nails were evaluated for whitening. The nail evaluators were unaware of the endpoints of the patients. To improve the diagnosis of white nails, the family physicians who performed the nail examinations in this study were trained using clinical pictures of white nails from a previous study (Figure 1) [5,6].

More than two physicians evaluated the participants to enhance the reliability of diagnoses. Furthermore, discrepancies between the assessors were resolved by discussion with another physician until an agreement was reached [10].

Covariates

The participants’ background information was obtained from the electronic health records of Unnan City Hospital. Participants’ data included age, sex, body mass index (BMI), and albumin (g/dL) concentration for nutrition assessment. Renal function was evaluated using serum creatinine and estimated glomerular filtration rate (eGFR). Hemoglobin concentration and care level based on the Japanese long-term insurance system were also evaluated [11]. In addition, the Charlson comorbidity index (CCI) based on patients’ medical histories was used to indicate the severity of their conditions. This included the presence of heart failure, myocardial infarction, asthma, chronic obstructive pulmonary diseases (COPD), kidney diseases, liver diseases, diabetes mellitus (DM), brain infarction, brain hemorrhage, hemiplegia, connective tissue diseases, dementia, and cancer [12]. Furthermore, therapists measured the cognitive and motor components and the functional independence measure (FIM) total score at discharge. These scores served as indicators of a patient’s daily activities and the places to which the patients were discharged (home or facility).

Statistical analysis

The Student’s t-test and the Mann-Whitney U test were used to analyze parametric and non-parametric data, respectively. Based on previous studies and the average of variables, numerical variables were dichotomized as follows: CCI (≥5 and <5) [12] and care level (≥1 and 0) based on the burden on caregivers and families [12]. Statistically significant factors in the univariate analysis and related factors in the previous study were entered into the multivariate analysis using the Cox proportional hazard regression model to determine independent predictors of readmission after discharge [13]. Cumulative event-free survival rates were determined using the Kaplan-Meier method and analyzed using the log-rank test. Statistical significance was defined as a p-value <0.05. In sample size determination, the calculated sample size for detecting a difference in the percentage of readmission of 10% between groups with and without white nails was 219 participants with 80% statistical power and 5% type 1 error. All statistical analyses were performed using EZR (Saitama Medical Center, Jichi Medical University, Saitama, Japan) and a graphical user interface for R (The R Foundation, Vienna, Austria).

Ethics approval

All participants were informed of the purpose of this research. Informed consent was obtained from all participants or their families. The Clinical Ethics Committee of Unnan City Hospital approved this research (approval code: 20200017).

## Results

Participant demographics

**Figure 2:**
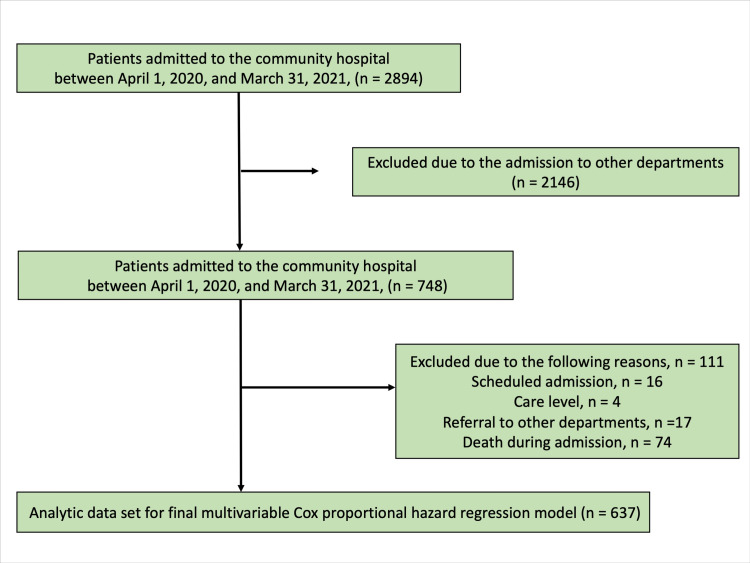
Flowchart of patient selection.

Of the 2,894 patients admitted to the community hospital, 748 were admitted to the Department of General Medicine, where trained family physicians examined the presence of white nails. After excluding patients with missing data, 637 patients were evaluated. A flowchart illustrating patients’ inclusion is presented in Figure 2.

Of the participants, 24.8% had white nails. The average age of the participants was 81.2 (standard deviation (SD) = 14.03) years, and 43.2% were men. Statistically significant differences were found between the group that included readmitted patients and the group who were not readmitted regarding albumin concentration, presence of white nails, time to readmission, home discharge, CCI, heart failure, peptic ulcer, DM, COPD, cancer, and dementia (Table 1).

**Table 1:** Patient demographics. BMI: body mass index; eGFR: estimated glomerular filtration rate; FIM: functional independence measure; CCI: Charlson comorbidity index; MI: myocardial infarction; COPD: chronic obstructive pulmonary disease; DM: diabetes mellitus; SD: standard deviation; IQR: interquartile range

		Readmission		
Factor	Total	Yes	No	P-value
N	637	183	454	
Age, mean (SD)	81.20 (14.03)	82.78 (13.00)	80.56 (14.39)	0.071
Male sex (%)	275 (43.2)	89 (48.6)	186 (41.0)	0.093
Albumin, mean (SD)	3.55 (0.64)	3.44 (0.63)	3.59 (0.65)	0.008
Height, mean (SD)	153.45 (10.51)	153.60 (9.69)	153.38 (10.83)	0.811
Weight, mean (SD)	49.18 (11.90)	48.42 (11.01)	49.48 (12.24)	0.309
BMI, mean (SD)	20.76 (3.96)	20.48 (4.06)	20.88 (3.91)	0.259
Creatinine	1.04 (1.12)	1.04 (0.88)	1.05 (1.21)	0.924
eGFR	58.86 (22.01)	57.20 (21.98)	59.53 (22.01)	0.227
Hemoglobin, mean (SD)	11.93 (4.28)	11.51 (2.41)	12.09 (4.83)	0.122
FIM score at discharge				
Total FIM score (median)	92.00 (18, 126)	90.00 (18, 126)	93 (18, 126)	0.491
Motor domain score (median)	63 (13, 91)	59.00 (13, 91)	65.00 (13, 91)	0.545
Cognitive domain score (median)	31 (5, 35)	31 (5, 35)	30.5 (5, 35)	0.848
White nail (%)	158 (24.8)	65 (35.5)	93 (20.5)	<0.001
Home discharge (%)	170 (26.9)	32 (17.5)	138 (30.7)	0.001
Duration of previous hospitalization, median (IQR)	15 (1, 532)	11 (1, 532)	16 (1, 390)	0.005
CCI ≥ 5 (%)	368 (57.8)	127 (69.4)	241 (53.1)	<0.001
CCI (%)				
1	43 (6.8)	8 (4.4)	35 (7.7)	
2	28 (4.4)	1 (0.5)	27 (5.9)	
3	48 (7.5)	12 (6.6)	36 (7.9)	
4	150 (23.5)	35 (19.1)	115 (25.3)	
5	132 (20.7)	30 (16.4)	102 (22.5)	
6	97 (15.2)	34 (18.6)	63 (13.9)	
7	80 (12.6)	30 (16.4)	50 (11.0)	
8	30 (4.7)	16 (8.7)	14 (3.1)	
9	19 (3.0)	11 (6.0)	8 (1.8)	
10	4 (0.6)	3 (1.6)	1 (0.2)	
11	2 (0.3)	2 (1.1)	0 (0.0)	
12	2 (0.3)	1 (0.5)	1 (0.2)	
13	1 (0.2)	0 (0.0)	1 (0.2)	
15	1 (0.2)	0 (0.0)	1 (0.2)	
Heart failure (%)	109 (17.1)	43 (23.5)	66 (14.5)	0.01
MI (%)	47 (7.4)	16 (8.7)	31 (6.8)	0.405
Asthma (%)	32 (5.0)	12 (6.6)	20 (4.4)	0.315
Peptic ulcer (%)	58 (9.1)	23 (12.6)	35 (7.7)	0.066
Kidney disease (%)	50 (7.8)	18 (9.8)	32 (7.0)	0.255
Liver disease (%)	24 (3.8)	5 (2.7)	19 (4.2)	0.493
COPD (%)	33 (5.2)	19 (10.4)	14 (3.1)	0.001
DM (%)	97 (15.2)	36 (19.7)	61 (13.4)	0.052
Brain infarction (%)	117 (18.4)	41 (22.4)	76 (16.7)	0.113
Brain hemorrhage (%)	46 (7.2)	15 (8.2)	31 (6.8)	0.612
Hemiplegia (%)	21 (3.3)	6 (3.3)	15 (3.3)	1
Connective tissue disease (%)	24 (3.8)	8 (4.4)	16 (3.5)	0.647
Dementia (%)	115 (18.1)	42 (23.0)	73 (16.1)	0.052
Cancer (%)	114 (17.9)	47 (25.7)	67 (14.8)	0.002
Dependent condition (%)	258 (40.5)	84 (45.9)	174 (38.3)	0.09
Care level (%)				
0	379 (59.5)	99 (54.1)	280 (61.7)	
1	47 (7.4)	15 (8.2)	32 (7.0)	
2	67 (10.5)	29 (15.8)	38 (8.4)	
3	63 (9.9)	23 (12.6)	40 (8.8)	
4	46 (7.2)	11 (6.0)	35 (7.7)	
5	35 (5.5)	6 (3.3)	29 (6.4)	

Association between the presence of white nails and other variables

Statistically significant differences were found between the groups with and without white nails with respect to age, albumin concentration, BMI, hemoglobin level, FIM score, home discharge, CCI, duration of the previous hospitalization, readmission, brain infarction, and dementia (Table 2).

**Table 2:** Association between the presence of white nails and other variables. BMI: body mass index; eGFR: estimated glomerular filtration rate; FIM: functional independence measure; CCI: Charlson comorbidity index; MI: myocardial infarction; COPD: chronic obstructive pulmonary disease; DM: diabetes mellitus; SD: standard deviation; IQR: interquartile range; +: presence of white nails; −: absence of white nails

	White nail		
Factor	+	-	P-value
N	158	479	
Age, mean (SD)	86.39 (10.21)	79.49 (14.69)	<0.001
Male sex (%)	61 (38.6)	214 (44.7)	0.195
Albumin, mean (SD)	3.05 (0.61)	3.71 (0.57)	<0.001
Height, mean (SD)	150.88 (9.78)	154.29 (10.61)	<0.001
Weight, mean (SD)	44.43 (10.97)	50.75 (11.79)	<0.001
BMI, mean (SD)	19.47 (4.24)	21.19 (3.77)	<0.001
Creatinine	1.02 (0.71)	1.05 (1.23)	0.734
eGFR	58.02 (26.19)	59.14 (20.47)	0.583
Hemoglobin, mean (SD)	10.86 (2.26)	12.28 (4.72)	<0.001
FIM score at discharge			
Total FIM score, median (IQR)	48.50 (18, 126)	102.00 (18, 126)	<0.001
Motor domain score, median (IQR)	25.50 (13, 91)	72.00 (13, 91)	<0.001
Cognitive domain score, median (IQR)	21.50 (5, 35)	35.00 (5, 35)	<0.001
Home discharge (%)	73 (46.5)	97 (20.4)	<0.001
Duration of previous hospitalization, median (IQR)	21 (1, 532)	13 (1, 390)	<0.001
Readmission (%)	65 (41.1)	118 (24.6)	<0.001
CCI ≥ 5 (%)	120 (75.9)	248 (51.8)	<0.001
CCI (%)			
1	4 (2.6)	39 (8.1)	
2	2 (1.3)	26 (5.4)	
3	3 (1.9)	45 (9.4)	
4	29 (18.4)	121 (25.3)	
5	40 (25.3)	92 (19.2)	
6	33 (20.9)	64 (13.4)	
7	24 (15.2)	56 (11.7)	
8	12 (7.6)	18 (3.8)	
9	7 (4.4)	12 (2.5)	
10	2 (1.3)	2 (0.4)	
11	1 (0.6)	1 (0.2)	
12	1 (0.6)	1 (0.2)	
13	0 (0.0)	1 (0.2)	
15	0 (0.0)	1 (0.2)	
Heart failure (%)	34 (21.5)	75 (15.7)	0.113
MI (%)	14 (8.9)	33 (6.9)	0.387
Asthma (%)	8 (5.1)	24 (5.0)	1
Peptic ulcer (%)	17 (10.8)	41 (8.6)	0.426
Kidney disease (%)	17 (10.8)	33 (6.9)	0.126
Liver disease (%)	6 (3.8)	18 (3.8)	1
COPD (%)	10 (6.3)	23 (4.8)	0.534
DM (%)	31 (19.6)	66 (13.8)	0.096
Brain infarction (%)	38 (24.1)	79 (16.5)	0.043
Brain hemorrhage (%)	8 (5.1)	38 (7.9)	0.288
Hemiplegia (%)	7 (4.4)	14 (2.9)	0.44
Connective tissue disease (%)	6 (3.8)	18 (3.8)	1
Dementia (%)	42 (26.6)	73 (15.2)	0.002
Cancer (%)	36 (22.8)	78 (16.3)	0.073
Dependent condition (%)	102 (64.6)	156 (32.6)	<0.001
Care level (%)			
0	56 (35.4)	323 (67.4)	
1	12 (7.6)	35 (7.3)	
2	20 (12.7)	47 (9.8)	
3	28 (17.7)	35 (7.3)	
4	28 (17.7)	18 (3.8)	
5	14 (8.9)	21 (4.4)	

Timing of readmission

In total, 41.1% of patients with white nails were readmitted, while 24.6% of patients without white nails were readmitted (Table 2). With respect to the timing of readmission, 28.7% of participants were readmitted to the hospital during the study period (Table 3).

**Table 3:** Days from the discharge to readmission among the group of readmissions.

		Total participants (N = 637)			Readmitted participants (N = 183)	
Days to readmission	Number readmitted	Percentage	Cumulated percentage	Percentage		Cumulated percentage
<30 days	79	12.4	12.4	43.2		43.2
30 to 90 days	62	9.7	22.1	33.9		77.1
91 to 180 days	33	5.2	27.3	18.0		95.1
>180 days	9	1.4	28.7	4.9		100

More than 75% of readmissions were within 90 days, and more than 95% were within 180 days.

Regression model results

**Figure 3:**
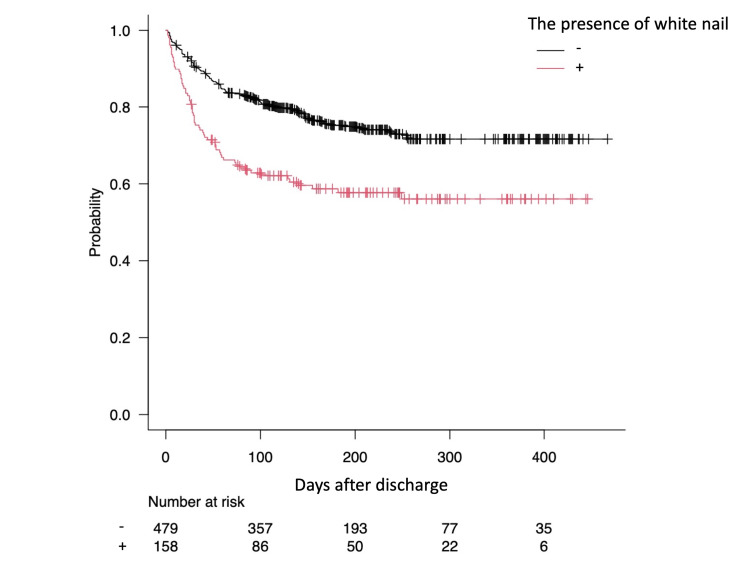
Kaplan-Meier curves showing the probability of readmission for patients who were previously discharged.

Kaplan-Meier curves showed the estimated probability of staying free of hospital readmission as a function of white nails (p < 0.001) (Figure 3).

Cox regression analysis was performed for age, sex, BMI, albumin, CCI ≥5, presence of cancer, COPD, dementia, DM, peptic ulcer, heart failure, home discharge, time to readmission, dependent condition, and white nails. The results are presented in Table 4.

**Table 4:** Results of the Cox regression model for hospital readmission. BMI: body mass index; CCI: Charlson comorbidity index; COPD: chronic obstructive pulmonary disease; DM: diabetes mellitus; CI: confidence interval

Factor	Hazard ratio	95% CI	P-value
Age	1	0.99–1.02	0.85
Sex	1.16	0.84–1.60	0.36
BMI	0.99	0.96–1.03	0.74
Albumin	0.83	0.63–1.09	0.18
CCI ≥ 5	1.20	0.79–1.82	0.40
Cancer	1.52	1.03–2.22	0.033
COPD	1.67	0.98–2.87	0.06
Dementia	1.52	1.03–2.25	0.037
DM	1.17	0.80–1.71	0.42
Peptic ulcer	1.14	0.72–1.83	0.57
Heart failure	1.53	1.06–2.21	0.025
Home discharge	0.34	0.22–0.52	<0.001
Duration of previous hospitalization	0.99	0.99–1.00	0.0026
Dependent condition	1.25	0.87–1.81	0.23
White nail	2.07	1.45–2.97	<0.001

Statistically significant variables with readmission were cancer (hazard ratio, HR = 1.52, p = 0.033), dementia (HR = 1.52, p = 0.037), heart failure (HR = 1.53, p = 0.033), home discharge (HR = 0.32, p ≤ 0.001), time to readmission (HR = 0.99, p = 0.0026), and presence of white nails (HR = 2.07, p ≤ 0.001).

Reasons for readmission to the hospital

Table 5 lists the diagnoses and frequencies for hospital readmission. The most frequent disease was pyelonephritis (18.0%), followed by heart failure (16.9%), pneumonia (12.6%), brain stroke (6.0%), and fracture (4.9%).

**Table 5:** Diagnoses and their frequencies among readmitted patients. Pneumonia includes bacterial, viral, and aspiration pneumonia. Brain strokes include brain infarction, brain hemorrhage, and transient ischemic attack. Fractures include femoral neck, pelvic, and vertebral fractures. Cancer includes colon, stomach, and pancreatic cancers. “Others” include upper gastrointestinal bleeding, prostatitis, pneumothorax, pleuritis, Parkinson’s disease, nephrotic syndrome, hyponatremia, gastroesophageal reflux disease, encephalitis, empyema, diverticulitis, rheumatoid arthritis, cholecystitis, cellulitis, asthma, aortic abscess, anaphylaxis, and abdominal hernia (n = 1, each category).

Diagnosis	Number	Percentage	Diagnosis	Number	Percentage
Pyelonephritis	33	18.0%	Ischemic colitis	3	1.6%
Heart failure	31	16.9%	Cholangitis	3	1.6%
Pneumonia	23	12.6%	Pseudomembranous colitis	3	1.6%
Brain stroke	11	6.0%	Syncope	3	1.6%
Fracture	9	4.9%	Hypoglycemia	3	1.6%
Epilepsy	8	4.4%	Medicine related	2	1.1%
Appetite loss	8	4.4%	Hypothyroidism	2	1.1%
Cancer	5	2.7%	Hydrocephalus	2	1.1%
Sepsis	4	2.2%	Hepatic encephalopathy	2	1.1%
Pseudogout	4	2.2%	Chronic obstructive lung disease	2	1.1%
Ileus	4	2.2%	Others	18	9.9%

## Discussion

This prospective cohort study showed that white nails were related to readmission after discharge from rural community hospitals among rural older patients. This effect persisted after adjusting for other independent variables related to hospital readmission. Cancer, dementia, and heart failure were positively associated with readmission to hospital among older people, in addition to white nails. Home discharge and time to readmission were negatively related to hospital readmission. The presence of white nails was related to various patient backgrounds.

Examining the whitening of nails can be emphasized when healthcare professionals examine older patients to consider the risk of readmission. This research showed that white nails can be related to hospital readmission among older, previously admitted patients. Older patients’ medical histories and nutritional conditions can affect various symptoms and physical findings, including nail findings [14]. In addition, nails can be quickly examined in comparison with other physical findings of the body, such as those related to the lungs, heart, and abdomen. Therefore, clinicians should consider older patients’ conditions comprehensively, including white nail findings [14].

This study shows that white nails may be related to patient readmission to hospitals due to these issues. The readmission of older patients to hospitals can be affected by nutrition, age, cognitive function, and previous clinical conditions [15]. Furthermore, this study showed that 77.1% of readmissions occurred within 90 days. Therefore, clinicians should be aware that patients with white nails present a higher risk of readmission. They should follow a clinical course with more effective collaboration with other healthcare professionals. The presence of white nails can be explained by conditions other than nutrition. Nail bed color can result from abnormalities in the vascular nail bed or substance deposition [16]. Nevertheless, future studies may clarify the pathophysiology of white nails.

Clinicians should consider various clinical factors related to the readmission of older admitted patients in community hospitals to effectively discharge older patients with white nails. Serum albumin levels may be associated with the readmission of older admitted patients. Serum albumin is related to infection, dehydration, and nutritional deficiency [17,18]. Low serum albumin may indicate poor nutritional conditions with the inability to secrete albumin in the liver or severe infections with an increase in immunoglobulin-suppressing albumin production, which can cause high mortality [19]. Diseases such as cancer, COPD, and heart failure can increase readmission rates, and the longevity of patients with these diseases may be lower in developed countries [20]. Thus, patients with cancer, COPD, or heart failure can be more vulnerable, even after being cured of acute diseases [21]. Appropriate preparation for home discharge should be established to reduce the chances of readmission.

During home discharge of patients with white nails, care should be provided by their caregivers and rural community healthcare workers, such as care managers, home care nurses, and home care workers, to enable effective home care. For home care of dependent patients, collaborative care is needed, utilizing available rural healthcare resources [22]. Patient preferences for health care can also be discussed and respected when they present with symptoms, which will lower readmission rates [23]. Thus, adequate preparation for discharge may need time to adjust homes or facilities to accommodate patients.

Preparation of effective discharge tends to necessitate more extended hospitalization. As this study showed, longer hospitalization was related to a lower probability of readmission. Preparation for home discharge needs interprofessional collaboration to prevent complications such as aspiration pneumonia, urinary tract infection, and heart failure [24]. In this study, the most common diseases leading to readmission among older people were pyelonephritis (18.0%), heart failure (16.9%), pneumonia (12.6%), brain stroke (6.0%), and fracture (4.9%). In aging societies, the prevalence of diseases within communities has changed, and the need for community hospitals is expanding. Adequate home care management can reduce the risk of readmission based on this research; however, the preparations may require extending the admission duration [25]. In rural contexts, practical preparation for discharge with adequate preparation time should be considered.

Whitening of the nails may be associated with various factors that should be investigated based on the pathophysiology. Based on this study, age, albumin concentration, BMI, and hemoglobin level were related to the presence of white nails, which can be explained by the process of aging and deterioration of nutritional conditions [26]. Poor activities of daily living (ADL) and severity of medical conditions may be associated with the presence of white nails, which may be related to the physiology of human bodies. Multiple diseases increase the inflammation rate, which may exacerbate atherosclerosis in the extremities [27]. This process may decrease perfusion to nails, triggering the whitening of nails. Another possibility is that less movement of extremities may induce whitening of nails. Patients with poor ADL have decreased usage of their extremities [28]. Less use of extremities may cause atrophy of these organs, including the arteries and veins [29]. Atrophy may decrease the perfusion of nails, triggering the whitening of nails. These hypotheses should be investigated in future research.

One limitation of this study was the study setting. The current societal aging rate in rural Japanese areas can differ from other countries’ rural areas. However, the study results can be used in countries with a high proportion of aged individuals. In addition, a low rate of missing data was achieved via diligent follow-up of all admitted patients, contributing to the reliability of the study’s findings. However, some patients might not have been followed up because of possible relocation to another city where they could not be transferred to the hospital; these situations may have affected the reliability of the findings. Another limitation was the diagnosis of white nails; hence, training to detect white nails could be needed. In this study, the images of white nails were used to educate family physicians. Simple definitions, such as the disappearance of the lunula and the whitening of the proximal part of the nail, can be important.

## Conclusions

White nails are related to the likelihood of readmission among older previously admitted patients in a rural context. Cancer, dementia, and heart failure are positively associated with hospital readmission. Pyelonephritis, heart failure, and pneumonia were the top three diseases associated with hospital readmission. Recognition of white nails may contribute to an awareness of the risk of readmission in older admitted patients. Information about assessing nails should be shared among caregivers and health providers, providing reasonable care with interprofessional collaboration. However, further research is required before use in clinical practice.
